# The Appointment and Conditions of Service of the Territorial Regimental Surgeon

**Published:** 1909-03-06

**Authors:** 


### The Royal Army Medical Corps Section.

### THE APPOINTMENT AND CONDITIONS OF SERVICE OF THE TERRITORIAL REGIMENTAL SURGEON.

In a recent article in The Hospital we drew attention to the very useful sphere of work that belongs to the Territorial regimental surgeon. As the position should have its attractions for the younger members of the medical profession, we propose in the present article to outline briefly the course of procedure to be followed for obtaining such an appointment, stating also the obligations that it entails.

### Procedure and Form of Application.

A candidate must be a registered qualified medical man, and the actual application for a Commission in the Territorial Medical Force must be made on a special form known as Army Form E 536, which can be obtained at the headquarters of any Territorial unit, or from the Secretary of any of the Territorial County Associations. This Army Form contains all the required details which the applicant has to furnish. These he fills in and signs, and he obtains also signatures to the certificates of health and moral character which are embodied in the Army Form. Having thus completed the latter he forwards it with a copy of his birth certificate to the principal medical officer of the division in which he desires to serve, indicating the particular unit or regiment to which he would like to be attached. On receipt of it the principal medical officer sends it with a letter of recommendation to the Secretary of the County Association that administers the unit the applicant has asked to be attached to. From the County Association the application passes to the general officer commanding the military district, and he in his turn transmits it to the Army Council, who signify that the application is granted by a formal announcement in the *London Gazette*, which officially intimates that a commission in the Royal Army Medical Corps (T.F.) has been granted to the applicant, and that he is to do duty with the particular unit he desired.

### Uniform, Training, and Camp.

The rank granted on appointment is that of lieutenant, and the uniform worn is that of the R.A.M.C. (T.F.). This latter comprises a service suit of khaki and the blue ceremonial dress with cherry facings that is worn on special occasions. The expenses of the uniform required should not exceed £40, and towards this a grant of £20 is given by the Government as soon as the officer has gone through his training course and has become what is termed "efficient." The conditions required for efficiency are different the first year from subsequent ones. For the former they consist of a minimum of thirty drills, of which fifteen must be put in before going to camp which is held annually. This attendance of fifteen drills preliminary to camp is important, as without them the medical officer is not eligible to draw pay while in camp. The annual training in camp must be for a period of not less than eight days, but can be extended to fifteen days, or eighteen days in the case of the Yeomanry medical officers. There is complete freedom of choice as to which period of camping is selected, but whether eight or fifteen days the regimental medical officer is paid for each day's residence in camp the sum of £1 2s. per diem, this amount including all allowances.

Attendance at camp even for eight days is not, however, compulsory, and if circumstances stand in the way leave of absence can be obtained from the principal medical officer of the division with which the medical officer is serving. If exemption is granted, then he must go through an alternative course at a Territorial School of Instruction, at a military or civil hospital, or other approved institution. The course extends over eight days (not necessarily consecutive) and is reckoned to comprise twenty-four attendances, of which not more than three can take place in one day, and each of them having a duration of not less than one hour. If the medical officer makes his eight days' course a consecutive one, then he is entitled to pay during it at the rate of 18s. per diem, the smaller amount being due to the fact that the field and furniture allowances given in camp are not granted. Should the medical officer have put in only fifteen drills before the annual camp, then he must attend the other fifteen before October 31, which closes what may be called the Territorial year, otherwise he will not be reckoned as efficient and consequently he will not be eligible to draw his £20 grant for uniform.

### Promotion and Pay.

The recognition of his efficiency takes the place of the Certificate A that has to be obtained by officers of other branches of the Territorial Force on their promotion from second lieutenant to rank of lieutenant; for the Territorial medical officer being gazetted lieutenant when he joins has no need for a promotion examination to that rank. For subsequent promotion he has, however, to qualify, and his training after his first year is to obtain Certificate B and qualify for the rank of captain. This Certificate B takes the place of the old proficiency examination of Volunteer days, which was conducted by a board of army medical officers in the various military districts. It was found that the standard of examination and the subjects of examination varied considerably in the different districts, but this cannot happen under the new Territorial regulations, which lay down distinctly the scope and subject matter of examination.

The subjects laid down for this Certificate are as follows: (1) War establishments; (2) organisation of field medical units; (3) the work of medical units in the field; and (4) sanitation and other duties in barracks, camp, and on the line of march. During the years of study for this Certificate B there are demanded of the medical officer fifteen annual attendances at drill and his presence at the annual camp, but he can obtain exemption from the latter and substitute for it the previously mentioned eight days' alternative course. Until the rank of captain is reached the same rate of pay per diem is given during camp or the alternative course as was allowed in the first year. After 3½ years' service the rank of captain is given to all those holding certificate B. This higher rank carries with it increased pay both in camp and during an alternative course, the amount paid to a captain being £1 4s. 6d. in camp and £1 1s. during an alternative course. A further service of 8½ years is necessary before the rank of major can be obtained, and to be eligible for it the regimental medical officer must repeat the eight days' course as an independent one, not alternative to camp. When appointed major the medical officer can continue to serve with his regimental unit, but if he aspires to the rank of lieutenant-colonel he must gain a further special certificate for this higher rank. If successful and he is gazetted lieutenant-colonel he must then leave his unit and is available for other duties.

Such is a brief outline of the conditions of service for the Territorial regimental medical officer, and we do not regard them as onerous or as making an unreasonable demand on any young medical man. They are framed in an accommodating spirit, and they recognise that the Territorialist is a civilian first and a soldier afterwards. We consider, too, that no medical man need be deterred from joining by fear of the position making heavy demands on his purse. Any necessary social outlays are moderate and entirely voluntary, while the expenses of camp can be easily met by the pay earned. A good deal might be said of the advantages that accrue from membership of the R.A.M.C. (T.F.). Camp life in the summer has many delights, and what we would not have forgotten is that the duties of the regimental surgeon complete in a very definite way the professional education of the medical man, especially in the subjects of first-aid and practical sanitation that the stereotyped medical curriculum is deficient in. Speaking generally the Territorial medical service by its discipline, its *esprit de corps*, its awakening of patriotism, and its healthy camp life makes those who join it bodily and mentally better men and more useful citizens.

